# Participation of Black US Residents in Clinical Trials of 24 Cardiovascular Drugs Granted FDA Approval, 2006-2020

**DOI:** 10.1001/jamanetworkopen.2021.2640

**Published:** 2021-03-23

**Authors:** Siliang Chen, Jiarui Li

**Affiliations:** 1 Department of Medical Oncology, Peking Union Medical College Hospital, Chinese Academy of Medical Sciences and Peking Union Medical College, Beijing, China

## Abstract

This cross-sectional study investigates the representation status of Black US residents in clinical trials of 24 cardiovascular drugs granted US Food and Drug Administration (FDA) approval between January 1, 2006, and August 31, 2020.

## Introduction

Although mortality from cardiovascular disease (CVD) has declined consistently in the United States, CVD remains a major public health burden for Black US residents.^1,2^ Given that clinical trials provide the foundational evidence that helps shape guideline recommendations for physicians and support approval of new drugs by the US Food and Drug Administration (FDA), equitable representation of Black US residents in clinical trials is vitally important. Therefore, this study was designed to investigate the representation status of Black US residents in crucial clinical trials supporting FDA approval of cardiovascular drugs.

## Methods

This cross-sectional study included 24 new molecular entity drugs for treatment of CVD approved by the FDA between January 1, 2006, and December 31, 2020. The analysis included 7 cardiovascular conditions: hypertension, coronary artery disease, acute coronary syndrome or myocardial infarction, heart failure, atrial fibrillation, pulmonary arterial hypertension, and hypercholesterolemia. Participation data for the clinical trials were derived from the Drugs@FDA database. Detailed data-gathering methods and calculations are described in the eMethods in the Supplement. The ethics committee of Peking Union Medical College Hospital determined that the study was exempt from institutional review board review due to the retrospective study design and use of deidentified data. This study followed the Strengthening the Reporting of Observational Studies in Epidemiology (STROBE) reporting guideline.

We evaluated the representation status of Black and White US residents in clinical drug trials by calculating the participation to prevalence ratio (PPR). A PPR between 0.8 and 1.2 indicates that the proportion of Black and White US residents in clinical trials nearly equals the proportion of participants of certain racial/ethnic groups in the disease population. Participation to prevalence ratios less than 0.8 or greater than 1.2 indicate that certain racial/ethnic groups are either underrepresented or overrepresented, respectively.

## Results

Between January 1, 2006, and December 31, 2020, the FDA approved 24 new molecular entity drugs for 7 cardiovascular conditions. In related clinical trials supporting FDA drug approval, a total of 187 294 participants were enrolled, including 5396 Black participants and 155 694 White participants (Table). For Black US residents, the percentage of clinical trial participation was 2.9%, and the total PPR for all CVD conditions was 0.29. The highest PPR for Black participants was 0.52 (hypertension), the lowest PPR was 0.072 (hypercholesterolemia), and all PPRs were less than 0.8, indicating underrepresentation (Figure, A). For White US residents, the percentage of clinical trial participation was 83.1%, and the total PPR was 1.14. The highest PPR for White participants was 2.77 (hypercholesterolemia), the lowest PPR was 0.88 (pulmonary arterial hypertension), and all PPRs were greater than 0.8 (Figure, A). The PPRs of White participants for acute coronary syndrome or myocardial infarction, coronary artery disease, heart failure, hypertension, and hypercholesterolemia were greater than 1.2, indicating overrepresentation. The breakdown of PPRs by year also showed underrepresentation of Black US residents (Figure, B).

**Table.** Participation by Black and White US Residents in Clinical Trials of Drugs to Treat CVD

Cardiovascular condition	No. of drug approvals	Drug (approval year)	Total trial enrollment, No.	Enrollment by race, No. (%)		CVD population by race, %		PPR by race	
				Black	White	Black	White	Black	White
Total	24	NA	187 294	5396 (2.9)	155 694 (83.1)	9.9	72.7	0.29	1.14
Acute coronary syndrome or myocardial infarction	3	Prasugrel hydrochloride (2009)	43 377	929 (2.1)	40 051 (92.3)	18.9	52.2	0.11	1.77
		Ticagrelor (2011)							
		Cangrelor (2015)							
Atrial fibrillation	5	Dronedarone hydrochloride (2009)	76 231	911 (1.2)	60 732 (79.7)	3.6	84.7	0.33	0.94
		Dabigatran etexilate mesylate (2010)							
		Rivaroxaban (2011)							
		Apixaban (2012)							
		Edoxaban tosylate (2015)							
Coronary artery disease	2	Ranolazine (2006)	28 028	708 (2.5)	24 621 (87.8)	19.0	50.0	0.13	1.76
		Vorapaxar sulfate (2014)							
Heart failure	2	Ivabradine hydrochloride (2015)	14 947	503 (3.4)	11 350 (75.9)	27.9	49.3	0.12	1.45
		Sacubitril- valsartan (2015)							
Hypertension	4	Aliskiren hemifumarate (2007)	15 255	2175 (14.3)	10 971 (71.9)	27.7	35.3	0.52	2.04
		Nebivolol hydrochloride (2007)							
		Clevidipine (2008)							
		Azilsartan kamedoxomil (2011)							
Pulmonary arterial hypertension	4	Ambrisentan (2007)	2992	71 (2.4)	1913 (63.9)	12.1	72.8	0.20	0.88
		Riociguat (2013)							
		Macitentan (2013)							
		Selexipag (2015)							
Hypercholesterolemia	4	Pitavastatin calcium (2009)	6464	99 (1.5)	6056 (93.7)	21.3	33.8	0.072	2.77
		Lomitapide mesylate (2012)							
		Mipomersen sodium (2013)							
		Bempedoic acid (2020)							

Abbreviation: CVD, cardiovascular disease; NA, not applicable; PPR, participation to prevalence ratio.

**Figure.**
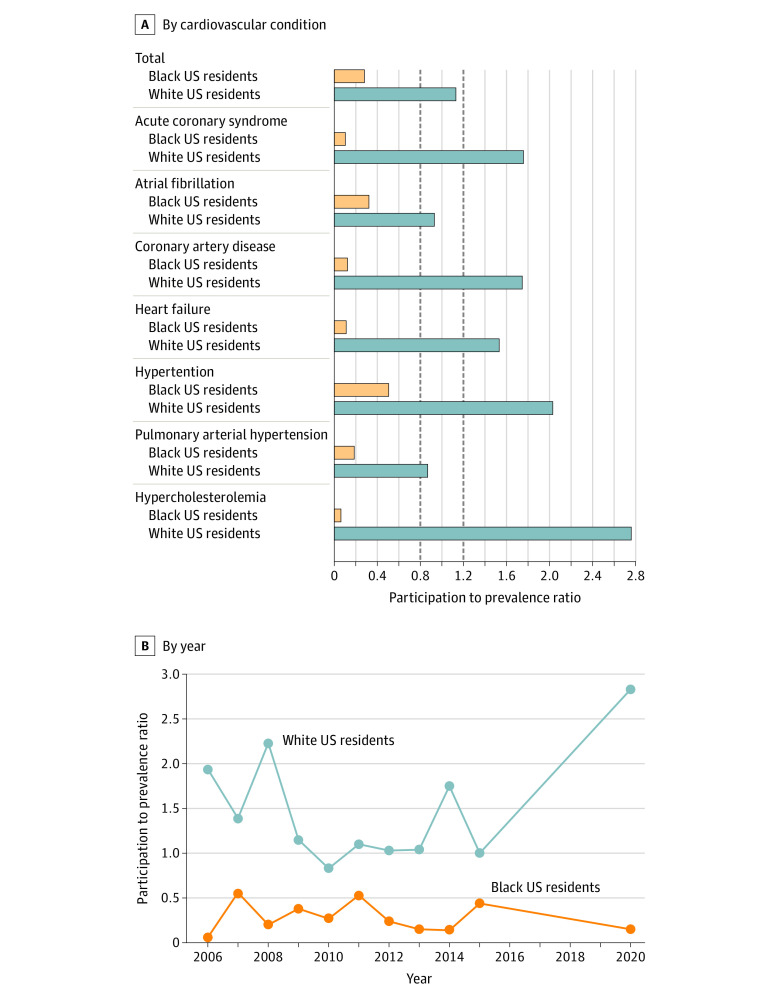
Participation by Black and White US Residents in Clinical Trials Supporting Approval of Drugs to Treat Cardiovascular Disease, 2006-2020 A, The vertical dashed line at participation to prevalence ratio (PPR) of 0.8 indicates that lower values represent underrepresentation, and the dashed line of 1.2 indicates that higher values represent overrepresentation. Values from 0.8 to 1.2 indicate that the proportion of the group in clinical trials nearly equals its proportion in the disease population. B, An annual breakdown shows underrepresentation by Black US residents in cardiovascular drug trials.

## Discussion

Our findings show that Black US residents were underrepresented as participants in clinical trials supporting FDA approval of cardiovascular drugs, whereas White US residents were equally represented and even overrepresented. This marked underrepresentation might undermine the generalizability of use of new CVD drugs in Black US residents. Limitations of this study include the inability to adjust confounding factors because of inaccessible original data for these trials.

In 1993, the National Institutes of Health established the Revitalization Act to ensure that racial minority groups are adequately enrolled in clinical trials with measures taken to enhance enrollment of Black participants. These measures included consideration of recruitment of minority groups in the site selection stage, provision of additional funds to increase recruitment rate of Black participants, the 2010 launch of the FDA Office of Minority Health and Health Equity, establishment of FDA Drug Trials Snapshots, and the National Institutes of Health mandate for race/ethnicity subgroup analysis in phase 3 trials. However, possible implicit biases, language barriers, disparities in socioeconomic status, and unique cultural practices left it unclear whether racial minority groups could benefit from these measures. Although substantial efforts have been made to reduce racial inequity in clinical trials participation, previous studies have indicated that the enrollment of Black US participants in CVD trials was still disappointing and that significant racial disparities have persisted over the past 15 years.^3,4^ More effective strategies (eg, a 10%-50% accrual rate of minority groups in clinical trials) are required to enhance the enrollment of Black participants in CVD clinical trials.^5^

## Notes

Supplement. **eMethods.** Sampling Methods **eReferences.**
