# Supplementary material for: Participation of Black US Residents in Clinical Trials of 24 Cardiovascular Drugs Granted FDA Approval, 2006-2020
Source: JAMA Netw Open. 2021 Mar 23;4(3):e212640. doi: 10.1001/jamanetworkopen.2021.2640 (PMC7988366; doi:10.1001/jamanetworkopen.2021.2640)
Supplement: Supplement. — eMethods. Sampling Methods eReferences. [file jamanetwopen-e212640-s001.pdf]

Supplemental Online Content

Chen S, Li J. Participation of Black US residents in clinical trials of 24 cardiovascular drugs granted FDA approval, 2006-2020. *JAMA Netw Open.* 2021;4(3):e212640. doi:10.1001/jamanetworkopen.2021.2640

eMethods. Sampling Methods

eReferences.

This supplementary material has been provided to give readers additional information about their work.

© 2021 Chen S et al. *JAMA Network Open*

eMethods. Sampling Methods

Participation data of clinical trials were extracted based on the Drugs@FDA database. For hypertension, coronary artery disease, acute coronary syndrome or myocardial infarction, heart failure, and hypercholesterolemia, the percentages of Black and White US residents in disease population were estimated based on the National Health and Nutrition Examination Survey (NHANES).[1] For atrial fibrillation and pulmonary arterial hypertension, the percentages of Black and White US residents in disease population were estimated based on the nationwide AnTicoagulation and Risk Factors In Atrial Fibrillation (ATRIA) study and the Registry to Evaluate Early and Long-term Pulmonary Arterial Hypertension Disease Management (REVEAL) study.[2,3] To evaluate the representation status of Black and White US residents, we calculated the participation to prevalence ratio (PPR) by the formula

$$\frac{\text{percentage of black or white Americans among trial participants}}{\text{percentage of black or white Americans among disease population}}.$$

In addition, because the information of participants involved in similar research relied on ClinicalTrials.gov reports, we validated the trials that has equivalent numbers reported in both the FDA database and ClinicalTrials.gov databases.

© 2021 Chen S et al. *JAMA Network Open*

eReferences

1. Centers for Disease Control and Prevention. National Center for Health Statistics. National Health and Nutrition Examination Survey (NHANES). https://www.cdc.gov/nchs/nhanes/. Accessed January 21, 2021.

2. Go AS, Hylek EM, Phillips KA, et al. Prevalence of diagnosed atrial fibrillation in adults: national implications for rhythm management and stroke prevention: the AnTicoagulation and Risk Factors in Atrial Fibrillation (ATRIA) Study. JAMA. 2001;285(18):2370-2375.

3. Frost AE, Badesch DB, Barst RJ, et al. The changing picture of patients with pulmonary arterial hypertension in the United States: how REVEAL differs from historic and non-US Contemporary Registries. Chest. 2011;139(1):128-137

© 2021 Chen S et al. *JAMA Network Open*
